# Impact of COVID infection on lung function test and quality of life 

**DOI:** 10.1038/s41598-023-43710-w

**Published:** 2023-10-12

**Authors:** Ming Ren Toh, Ying Rachel Teo, Li Choo Ruby Poh, Yiting Tang, Rui Ya Soh, Kiran Sharma, Ganesh Kalyanasundaram, Kai Chin Poh

**Affiliations:** 1 Department of Clinical Measurement Centre, Sengkang General Hospital, Singapore, Singapore; 2 Department of Internal Medicine, Sengkang General Hospital, Singapore, Singapore; 3 Department of Respiratory and Critical Care Medicine, Singapore General Hospital, Singapore, Singapore; 4 Department of Respiratory Medicine, Sengkang General Hospital, Singapore, Singapore

**Keywords:** Diseases, Health care

## Abstract

Post-COVID-19 pulmonary sequalae are well-recognized early in the pandemic. Survivorship clinics are crucial for managing at-risk patients. However, it is unclear who requires pulmonary function test (PFT) and when PFTs should be performed. We aim to investigate for whom and how these interval PFTs should be performed. We performed a single-centre, prospective cohort study on COVID-19 survivors between 1st May 2020 and 31st April 2022. These patients were followed up at 6, 9 and 12 months with interval PFT and Short Form-36 (SF-36) Health Survey. Those with PFT defects were offered a computed tomography scan of the thorax. Of the 46 patients recruited, 17 (37%) had severe/critical illness. Compared to those with mild/moderate disease, these patients were more likely to experience DLCO defects (59% versus 17%, *p* = 0.005) and had lower SF-36 scores (mean physical component summary score of 45 ± 12 versus 52 ± 8, *p* = 0.046). These differences were most notable at 6 months, compared to the 9- and 12-months intervals. DLCO defects were also associated with older age, raised inflammatory markers and extensive CXR infiltrates. Besides interstitial-like abnormalities, obesity and undiagnosed lung conditions accounted for 39% of the PFT abnormalities. Interval PFTs can be performed earliest 6 months post-COVID-19. Patients with normal tests were unlikely to develop new abnormalities and would not require repeat PFTs. Abnormal PFTs can be followed-up with repeat PFTs 6 monthly until resolution. Non-COVID-19 differentials should be considered for persistent PFT abnormalities.

## Introduction

As of June 2023, over 700 million cases of Coronavirus Disease 2019 (COVID-19) and almost 7 million COVID-19 deaths have been recorded globally^1^. Among those who were hospitalized, 30–50% had severe disease leading to ICU admissions or death^2–4^. 20–60% of COVID-19 survivors can have respiratory symptoms, such as cough and dyspnoea, beyond the initial 12 weeks from COVID-19 infection^5–7^. The persistence of respiratory symptoms may be due to the development of physiological and structural lung abnormalities; a meta-analysis on post-COVID-19 pulmonary function and radiological abnormalities reported persistent diffusion limitations and fibrotic changes in almost one-third of the COVID-19 survivors 12 months after infection^8^. 30–70% of COVID-19 survivors also experience diminished mental well-being and quality of life (QOL)^5–7^.

Post-COVID-19 pulmonary sequalae has been well recognized early in the pandemic. Survivorship clinics were crucial for managing at-risk patients, especially those discharged from intensive care unit (ICU). Beyond these ICU survivors, it was not clear who else would benefit from pulmonary function evaluation and how long they should be followed up. Current understanding of post-COVID-19 pulmonary sequalae was limited by the considerable heterogeneity in study design, population selection, disease severity classification, measurement and reporting of results^8,9^. Most studies did not preclude patients with underlying lung diseases, which could result in over-estimation of COVID-19 associated pulmonary abnormalities^8^. There were also significant variations in lung function test procedures and diagnostic criteria, as well as radiological definitions of pulmonary fibrosis^10^. Often, pulmonary fibrosis was stated without specific description of the extent or radiological features of fibrosis^9^.

More follow-up studies were needed to understand the true burden of post-COVID-19 pulmonary sequalae and to optimise survivorship programs. To this end, we followed up a cohort of COVID-19 survivors, irrespective of their disease severity, and studied their temporal changes in lung function and QOL.

## Methods

We performed a prospective study on COVID-19 patients who were followed up at a COVID-19 survivorship clinic between 1st May 2020 to 31st April 2022. These patients were previously hospitalised for COVID-19 infection and referred to the clinic if they had residual respiratory symptoms at the time of discharge or had chest radiograph abnormalities due to COVID-19. We excluded pregnant women, patients with recent myocardial infarction, uncontrolled hypertension (> 180/100 mmHg), cognitive impairment and inability to understand the instructions for pulmonary function tests in English. Eligible patients were followed up at 6, 9 and 12 months with pulmonary function tests and Short Form-36 (SF-36) Health Survey.

Electronic records of the participants were retrieved for the patient’s demographics, clinical presentation, biochemical and radiological results, including those of chest X ray (CXR) and CT thorax (where applicable). We categorised COVID-19 into mild, moderate, severe and critical illness as per the National Institutes of Health (NIH), and dichotomised them into two severity groups for analysis (mild/moderate and severe/critical illness)^11^.

Pulmonary function tests (PFTs) included spirometry, single breath hold carbon monoxide uptake test and body plethysmography. The tests were performed using Platinum Elite™ Series from MCG Diagnostics Corporation, St Paul Minnesota USA. The following parameters were measured: forced vital capacity (FVC), forced expiratory volume in first second (FEV1), total lung capacity (TLC), residual volume (RV), diffusion capacity of the lung for carbon monoxide (DLCO), alveolar volume (VA) and carbon monoxide transfer coefficient (KCO). Pulmonary function tests were carried out in accordance with the European Respiratory Society-American Thoracic Society (ERS-ATS) guidelines and all parameters were recorded in standard international system of units (SI) units^12^. Reference values for spirometry and DLCO were obtained from the Global Lung Initiative (ERS) while those of TLC and RV were derived based on equations from Crapo et al.^13^. As per the ATS guidelines, impaired DLCO was defined as DLCO below the lower limit of normal (LLN), while a restrictive pattern was considered when TLC value was below LLN^14^. An obstructive pattern was considered when FEV1/FVC was below LLN, with FVC above LLN^14^.

The SF-36 Health Survey is an assessment of eight different health domains: physical function, social function, role limitation due to physical problems, role limitation due to emotional problems, mental health, bodily pain, vitality and general health^15^. Each domain is measured based on a score from 0 (worst) to 100 (best). Physical component summary (PCS) and mental component summary (MCS) scores were derived using the formulas by Thumboo et al. for the local population^16^.

Patients with persistent respiratory symptoms, unresolved CXR changes or pulmonary function tests abnormalities were advised to undergo evaluation with a computed tomography (CT) scan of the thorax. This involves a non-contrasted high resolution computed tomography (HRCT) sequence followed by a contrasted pulmonary embolism protocol sequence. CT scans of the thorax were performed on one type of scanner (Siemens SOMATOM Force: CARE Dose4D, Br40 kernel, IR = ADMIRE_3) and the CT images were constructed at 1 mm thickness and increment of 5 mm for lung sequence and at 3 mm thickness with increment of 3 mm for soft tissue sequence. Interstitial lung abnormalities (ILAs), as specified by the Fleischner Society, are non-dependent lung abnormalities on CT Thorax, including affecting more than 5% of any lung zone^17^. They include ground-glass opacities (GGOs), band-like reticulations, interlobular septal thickening, architectural distortion traction bronchiectasis and honeycombing.

### Statistical analysis

Descriptive statistics included means ± standard deviation and frequency as appropriate. Clinical characteristics of patients with mild/moderate and severe/critical COVID-19 infections were compared using independent t tests and chi-square tests for continuous and categorical variables respectively. Changes in pulmonary function and QOL with time and disease severity were analysed using linear mixed effects models, with random person intercept. Univariate and multivariate logistic regression were used to evaluate variables associated with diffusion defect (DLCO < LLN) at 6 months post-infection. Statistical analyses were performed using SPSS Statistics Version 16.0. All statistical tests were two tailed and a *p* value < 0.05 is denoted as statistically significant.

### Ethics statement

This study was approved by the Singhealth Centralised Institutional Review Board (CIRB Ref: 2020/2733) and all research was performed in accordance with relevant guidelines/regulations. Informed consent was obtained from all patients prior to pulmonary function testing.

## Results

**Figure 1:**
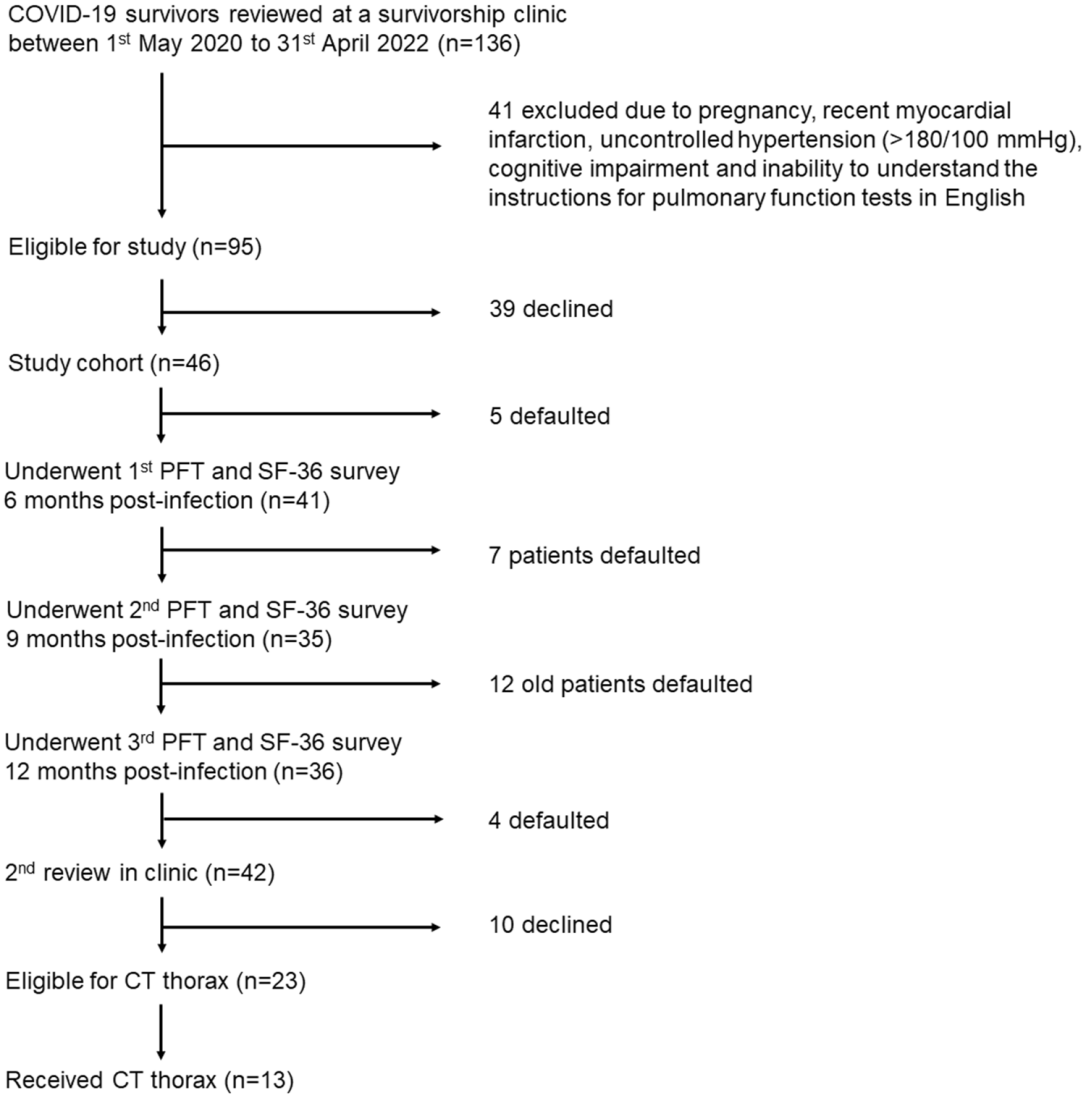
Study flowchart.

We recruited 46 patients for the follow-up lung function tests and SF-36 surveys (Fig. 1). 44 patients were infected with the Delta strain prior to 1st January 2022, and two patients were infected with the Omicron strain (after 1st January 2022). Mean age of the participants was 52 (± 14) years, with the majority being males (80%). Most patients were never smokers (74%) and did not have underlying chronic lung disease (87%). Four patients had asthma, 1 had chronic obstructive pulmonary disease and 1 had obstructive sleep apnoea (Table 1).

**Table 1:** Baseline characteristics.

	Mild or moderate infection, n = 29	Severe infection or critical illness, n = 17	*p* value
Age, years (mean ± SD)	47 ± 13	61 ± 11	< 0.001
Gender			
Female	6 (21%)	3 (18%)	0.561
Male	23 (79%)	14 (82%)	
BMI, kg/m^2^ (mean ± SD)	29 ± 10	29 ± 5	0.941
Smoking status			
Never smoker	22 (76%)	12 (71%)	0.476
Ex or current smoker	7 (24%)	5 (29%)	
Lung disease			
No	25 (86%)	15 (88%)	0.312
Asthma / Chronic obstructive pulmonary disease	4 (14%)	1 (6%)	
Obstructive sleep apnoea	0 (0)	1 (6%)	
Hypertension			
No	21 (72%)	6 (35%)	0.015
Yes	8 (28%)	11 (65%)	
Diabetes mellitus			
No	23 (79%)	8 (47%)	0.028
Yes	6 (21%)	9 (53%)	
White blood count (in 10^9^/L)			
4–10	25 (86%)	9 (53%)	0.018
< 4	4 (14%)	5 (29%)	
> 10	0	3 (18%)	
Platelet count (in 10^9^/L)			
150–450	26 (90%)	13 (77%)	0.216
< 150	3 (10%)	4 (23%)	
CRP (in mg/L)			
≤ 9.1	14 (54%)	1 (6%)	0.001
> 9.1	12 (46%)	16 (94%)	
Not done	3	0	
Procalcitonin (in mg/L)			
< 0.5	9 (47%)	0 (0)	0.001
≥ 0.5	10 (53%)	17 (100%)	
Not done	10	0	
Ferritin (in µg/L)			
≤ 339	4 (67%)	0 (0)	0.008
> 339	2 (33%)	10 (100%)	
Not done	23	7	
CXR infiltrates			
No	23 (79%)	2 (12%)	< 0.001
< 50% of lung fields	6 (21%)	6 (35%)	
≥ 50% of lung fields	0 (0)	9 (53%)	
Presence of pulmonary embolism			
No	29 (100%)	15 (88%)	0.131
Yes	0 (0)	2 (12%)	
Antiviral use			
No	27 (93%)	0 (0)	< 0.001
Lopinavir/ritonavir	0 (0)	5 (29%)	
Remdesivir	2 (7%)	12 (71%)	
Dexamethasone use			
No	27 (93%)	5 (29%)	< 0.001
Yes	2 (7%)	12 (71%)	
Anticoagulation use			
No	25 (96%)	5 (33%)	< 0.001
Yes	1 (4%)	10 (67%)	
Ventilatory support on admission			
None	29 (100%)	7 (41%)	< 0.001
NIV	0 (0)	2 (12%)	
HFNC	0 (0)	2 (12%)	
IMV	0 (0)	6 (35%)	
Antibiotic use for pneumonia			
No	24 (83%)	5 (29%)	< 0.001
Yes	5 (17%)	12 (71%)	

*BMI* body mass index, *CRP* c-reactive protein, *CXR* chest X-ray, *HFNC* high flow nasal cannula, *IMV* invasive mechanical ventilation, *NIV* non-invasive ventilation, *SD* standard deviation.

17 patients (37%) fulfilled the criteria for severe (n = 5) or critical illness (n = 12). Patients with severe/critical illness were more likely to have biochemical derangements in white cell, platelets, C-reactive protein, procalcitonin and ferritin (Table 1). Patients with severe/critical illness took longer for CXR resolution (mean 131 vs 7 days) compared to those with mild/moderate illness (Table 2).

**Table 2:** Pulmonary function test, SF-36 scores and radiological changes based on disease severity.

	Mild or moderate infection, n = 29	Severe infection or critical illness, n = 17	*p* value
Impaired DLCO			
No	24 (83%)	7 (41%)	0.005
Yes	5 (17%)	10 (59%)	
Restrictive lung defect			
No	23 (79%)	10 (59%)	0.126
Yes	6 (21%)	7 (41%)	
Obstructive lung defect			
No	26 (90%)	17 (100%)	0.241
Yes	3 (10%)	0 (0)	
MCS score 6 months post-infection (mean ± SD)	53 ± 7	49 ± 8	0.101
PCS score 6 months post-infection (mean ± SD)	52 ± 8	45 ± 12	0.046
Resolution of CXR changes			
Initial CXR normal	23 (82%)	1 (6%)	< 0.001
No	1 (4%)	7 (44%)	
Yes	4 (14%)	8 (50%)	
Not done	1	1	
Time taken for radiological resolution, days (mean ± SD)	8 ± 28	133 ± 152	< 0.001
Follow-up CT thorax			
Not indicated	21	2	
Indicated, not performed	6	4	
ILA absent	2 (100%)	2 (18%)	0.077
ILA present	0	9 (82%)	

*CXR* chest X ray, *DLCO* diffusion capacity of the lung for carbon monoxide, *ILA* interstitial lung abnormalities, *MCS* mental component summary, *PCS* physical component summary.

**Figure 2:**
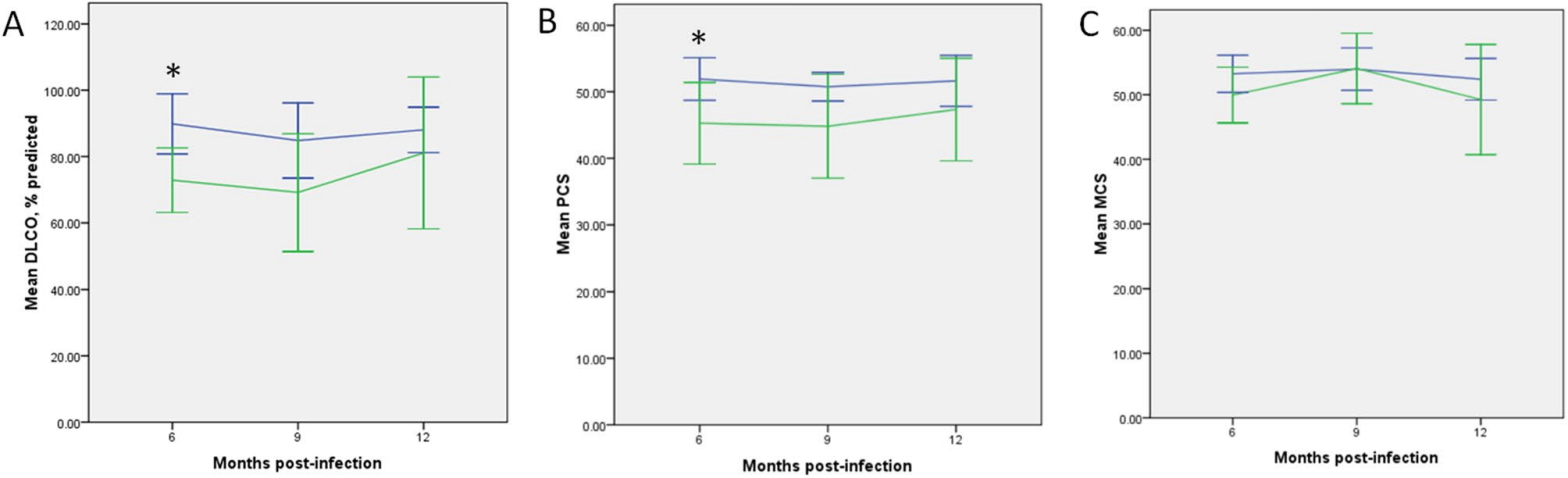
Plot of disease severity and time based on linear mixed model analysis. At 6 months post-infection, survivors of severe/critical COVID-19 had significantly lower DLCO and PCS scores compared to those with mild/moderate disease. These differences were not observed 9- and 12-months post-infection. Regardless of disease severity, there were no temporal difference in both pulmonary function and the SF-36 scores. Mild/moderate disease and severe/critical illness were represented by the blue and green lines respectively. Error bars indicated 95% confidence interval and statistical significance was represented by an asterisk. Abbreviations: DLCO (diffusing capacity of the lungs for carbon monoxide), MCS (mental component summary), PCS (physical component summary).

The most common PFT change was impaired DLCO (n = 15), followed by restrictive (n = 13) and obstructive (n = 3) ventilatory defects. These abnormalities occurred in 23 patients, with 10 patients having concomitant DLCO and restrictive ventilatory defects (Supplemental Table 1). Most abnormalities were detected at the first PFT (Table 3). Patients with normal PFTs at 6 months post-infection did not develop any abnormalities in the subsequent PFTs. Patients with severe/critical illness were more likely to have DLCO defects, compared to those with mild/moderate diseases (Table 2). They also had lower PCS and MCS scores, with PCS score at 6 months post-infection reaching statistical significance (Table 2). For both severity groups, there was no significant change in both DLCO and SF-36 scores over the 12-month period (Table 3, Fig. 2). Notably, 8 patients with DLCO defects underwent another PFT 18-months post-infection (outside of the study design), of which half achieved a normal DLCO (Supplemental Table 1).

**Table 3:** Pulmonary function test and SF-36 scores over time, regardless of disease severity.

	6 months (n = 41)	9 months (n = 35)	12 months (n = 36)	*p* value
DLCO defect	13	9	7	0.459
Restrictive defect	11	9	9	0.985
Obstructive defect	1	2	1	0.726
FVC, % predicted	94 ± 18	94 ± 17	93 ± 18	0.932
FEV1, % predicted	95 ± 18	94 ± 17	95 ± 18	0.983
DLCO, % predicted	84 ± 22	79 ± 27	86 ± 26	0.522
TLC, % predicted	89 ± 16	90 ± 16	89 ± 17	0.966
RV, % predicted	91 ± 24	92 ± 26	84 ± 32	0.443
MCS	52 ± 7	54 ± 8	51 ± 11	0.375
PCS	49 ± 10	49 ± 9	50 ± 11	0.871

*FVC* forced vital capacity, *FEV1* forced expiratory volume in 1 s, *DLCO* diffusing capacity of the lungs for carbon monoxide, *TLC* total lung capacity, *RV* residual volume, *MCS* mental component summary, *PCS* physical component summary.

DLCO defect was associated with age, raised inflammatory markers and extensive CXR infiltrates (> 50%) (Table 4). Patients with DLCO defects were also more likely to present with severe disease complicated by acute respiratory distress syndrome (ARDS) requiring ventilatory support (Table 4). At 6 months post-infection, these patients had a lower MCS score than those with normal DLCO (Table 4).

**Table 4:** Univariate analysis of factors associated with impaired DLCO.

	DLCO > LLN (n = 31)	DLCO < LLN (n = 15)	*p* value
Age, years (mean ± SD)	48 ± 13	61 ± 11	0.003
Gender			
Female	6 (19%)	3 (20%)	0.624
Male	25 (81%)	12 (80%)	
BMI, kg/m^2^ (mean ± SD)	29 ± 10	29 ± 5	0.873
Severity of illness			
Mild/moderate	24 (77%)	5 (33%)	0.005
Severe/critical	7 (23%)	10 (67%)	
ARDS			
No	27 (87%)	8 (53%)	0.018
Yes	4 (13%)	7 (47%)	
Ventilatory support			
No	27 (87%)	9 (60%)	0.047
Yes	4 (13%)	6 (40%)	
CRP			
Not available	3	0	
≤ 9.1	14 (50%)	1 (7%)	0.004
> 9.1	14 (50%)	14 (93%)	
Procalcitonin (in mg/L)			
Not available	9	1	
< 0.5	9 (41%)	0	0.005
≥ 0.5	13 (59%)	14 (100%)	
Ferritin (in µg/L)			
Not available	22	8	
≤ 339	3 (33%)	1 (14%)	0.392
> 339	6 (67%)	6 (86%)	
CXR infiltrates			
No	22 (71%)	3 (20%)	0.004
< 50% of lung fields	6 (19%)	6 (40%)	
≥ 50% of lung fields	3 (10%)	6 (40%)	
Resolution of CXR changes			
No interval CXR	1	1	
Initial CXR normal	21 (70%)	3 (21%)	0.010
No	5 (17%)	7 (50%)	
Yes	4 (13%)	4 (29%)	
Interval CT thorax			
Not done	27	6	
ILA absent	1 (25%)	3 (33%)	0.646
ILA present	3 (75%)	6 (67%)	
MCS score 6 months post-infection (mean ± SD)	54 ± 7	47 ± 7	0.007
PCS score 6 months post-infection (mean ± SD)	50 ± 9	48 ± 10	0.533

*ARDS* acute respiratory distress syndrome, *CXR* chest X ray, *CRP* c-reactive protein, *CT* computed tomography, *ILA* interstitial lung abnormalities, *MCS* mental component summary, *PCS* physical component summary.

Thirteen patients underwent a CT thorax during the study period (Fig. 1). None of the patients had overt fibrotic radiological abnormalities of volume loss, honeycombing and traction bronchiectasis. There were also no cases of pulmonary embolism. Among those patients with DLCO defects and corresponding evaluation with CT imaging (n = 9), the majority (78%) had subpleural bands, reticulations and GGOs which might explain their DLCO defects (Table 4). Besides non-fibrotic ILA, morbid obesity was another contributing cause of DLCO defects in 5 patients (Supplemental Table 1).

## Discussion

Patients with severe/critical COVID-19 infections should be considered for follow-up PFT, especially if they had more extensive systemic inflammation and radiological changes at presentation. In comparison, patients with mild infections did not have any PFT abnormalities or residual CXR changes and usually would not require follow-up PFT. We found that DLCO defects were the most common PFT abnormalities, consistent with studies performed in other centres where DLCO defects constituted 20–45% of the PFT abnormalities^8,18,19^. These defects could arise from the loss of ventilated alveolar units, alveolar membrane damage or microvascular abnormalities^20^. Patients with severe COVID-19 may experience delayed or absent initial adaptive immune response, leading to uncontrolled viral replication which triggers a cytokine storm with extensive pneumocyte injury and endothelial cell damage^21^. Hence, patients with severe COVID-19 were more susceptible to alveolar and endothelial injury and were more likely to develop DLCO defects than those with less severe disease^22^. This trend was observed among COVID-19 survivors across different ethnicities^22–24^. We also found that elderly patients were more likely to have DLCO defects, possibly due to the age-related attenuation in T cell response^23^.

Restrictive ventilatory defects were seen in 28% of our patients, similar to the prevalence reported in literature (8–20%)^8,24^. None of our patients with restrictive ventilatory defects had pulmonary fibrosis with volume losses. We hypothesised that obesity could have contributed partly to the restrictive and DLCO defects observed in our study, overestimating the true prevalence of COVID-19 related PFT changes. Extrapulmonary causes, such as obesity, respiratory muscle fatigue and localised microvascular changes have been frequently cited as causes of restrictive defects^20,25^. For example, a multi-centre study on post-COVID-19 PFTs cited obesity as a possible explanation for the impaired DLCO and FVC in almost 80% of the cases^25^.

We observed obstructive ventilatory defects in 3 patients and diagnosed one of them with asthma due to a positive methacholine challenge (Supplemental Table 2). Obstructive PFTs identified in COVID-19 survivorship clinics were usually due to other lung conditions such as asthma and chronic obstructive pulmonary disease (COPD)^22,26^. Hence, the detection of obstructive PFT patterns in COVID-19 survivors should be interpreted with caution and prompt further workup for an underlying chronic lung condition with the initiation of appropriate therapies (e.g. inhalers).

Overall, the true prevalence of COVID-19-related PFT abnormalities might have been overestimated by the underlying pulmonary and extrapulmonary conditions^22,25^. In the present study, 9 of the 23 patients with PFT abnormalities had undiagnosed asthma, COPD or were morbidly obese. Hence, future studies on COVID-19 related PFT changes should consider excluding patients with chronic lung conditions and account for the extrapulmonary causes such as obesity.

### Timing of PFT

Patients with persistent CXR abnormalities at 12 weeks post-infection should be considered for PFT based on the British Thoracic Society guidelines^27^. However, the duration and interval were not specified. Performing the PFT too early post-infection might overestimate the prevalence of COVID-19 pulmonary sequelae and lead to unnecessary follow-ups and testing.

We noted a lower proportion of abnormal PFT at 6 months post-infection (28%), than other studies which performed PFT at 1–3 months post infection (40–50%)^22,28^. Early PFT abnormalities may be due to post-infection interstitial and alveolar injury, and would not be representative of the chronic pulmonary sequelae, considering biological and physiological recovery can occur over months following the acute infection^29^.

In a single-centre study on 85 patients with non-critical COVID-19 patients (not requiring mechanical ventilation or ICU care), DLCO values were the lowest at time of discharge and recovered over time, albeit marginally from 80 to 86%^30^. Another single-centre study on 83 patients with severe COVID-19 showed that the predicted DLCO rose from 77 to 88%, between 3 and 12 months post-infection^31^. A meta-analysis on post-COVID PFT changes also reported a lower prevalence of impaired DLCO in the studies with a 12 month follow-up (31%), compared to those with a 6 month follow-up (39%)^8^. Lastly, previous studies on non-COVID-19 ARDS also showed a steady increase in DLCO and spirometry measures over time; DLCO recovery may lag behind those of FEV1 and FVC, sometimes normalising only after 5 years post-ARDS^32,33^.

Though there were no statistically significant PFT improvements over the 12 months study period, we observed that half of the DLCO defects due to COVID-19 had normalised by 18 months post-infection (Supplemental Table 1). Hence, we proposed that patients at risk of pulmonary sequalae can receive their initial PFT at 6 months post-infection with repeat PFT at 6 months interval (considering the recovery of DLCO defects might only occur 12–18 months post-infection).

### Radiological sequalae

Most of our study patients with mild/moderate disease achieved complete resolution of their presenting CXR changes. Patients who received a CT scan, mostly those with severe disease and DLCO defects, did not have any fibrotic changes. It is known that COVID-19 survivors with mild/moderate disease seldom sustain significant structural lung abnormalities^34,35^. Patients with more severe infections and ARDS may sustain long-term ILAs, though these were mostly mild non-fibrotic GGOs and/or reticulations (38–48%)^31,36–38^. Fibrotic changes were rare (10–12% of all post-COVID-19 ILAs) and localised (involving less than 25% of the lung parenchyma)^31,36–38^. These fibrotic ILAs can be of limited clinical significance; less than 2% of the patients with fibrotic ILAs and DLCO defects were symptomatic or dyspnoeic^39^. Thus, CT imaging should only be considered in patients with severe disease when there is high suspicion for pulmonary fibrosis or pulmonary embolism as the alternative cause for persistent symptoms or DLCO defects.

Like DLCO defects, ILA may resolve over time. A meta-analysis on the CT abnormalities following COVID-19 reported a lower prevalence of ILAs over time (39% at 6 months compared to 31% at 12 months follow-up), though this did not reach statistical significance^8^. The association between reticulation changes and DLCO defects had also been shown to attenuate over time^40^. Hence, we postulated that biological recovery from COVID-19 likely occurred in the majority of patients in the first few months after illness, and might continue beyond one year in patients with residual defects.

### Impact of COVID-19 on QOL

Besides the pulmonary and structural defects, COVID-19 infection could adversely impact patient’s QOL both during and after the infection^24^. We found that patients with more severe disease had lower PCS and MCS scores compared to those with milder diseases, consistent with the literature where COVID-19 survivors with severe infections had worse and more sustained QOL impairments compared to those with mild infections^41^. Patients with critical illness who were admitted to ICU showed the worst QOL indices, as part of the post-intensive care syndrome^42^. Patients with more comorbidities (including hypertension, diabetes, chronic lung disease) and higher BMI had a poorer QOL post-infection, in particular, lower PCS scores^41,43,44^. Similar to the present study, previous studies showed that patients with impaired DLCO had a worse QOL regardless of the disease severity^24,45^. Hence, patients with these risk factors should be identified early and followed up closely. They should be considered for early review by pulmonary rehabilitation and be referred for psychological support as required; previous studies have shown that early pulmonary rehabilitation can improve the dyspnoea, QOL and exercise capacity of patients with long COVID^46,47^.

### Strengths

Our study followed up COVID-19 survivors over regular intervals with repeat PFTs. Globally, there were few similar studies, considering the costs and time needed to perform PFTs which would be prohibitive especially amidst the COVID-19 pandemic. Our study findings could serve as a guidance for clinicians to decide on when and whom to perform PFTs. Moreover, we defined abnormal pulmonary function based on the LLNs, which has been shown to be less prone to misclassifications especially in older populations^12^. We also adopted a clear definition on the ILAs based on the Fleischner Society guidelines, to avoid misinterpreting terminologies and overestimating the prevalence of pulmonary fibrosis.

### Limitations

We had a small sample size which limited the statistical strength of our results and conclusions. The failure to observe statistically significant differences in DLCO and SF-36 between the two severity groups at 9 and 12 months could be caused by the higher attrition rates at these intervals. Patients who completed the entire study period were likely to be more health conscious, giving rise to selection bias. Nonetheless, our major findings were like those of other larger studies; for example, DLCO defect was the commonest PFT abnormality and the associated risk factors were age, disease severity and the degree of systemic inflammation. Lastly, CT imaging was not done in almost half of the indicated cases due to patient refusal. This further limited our analysis of the prolonged COVID-19 radiological sequalae.

## Conclusion

Most COVID-19 survivors with mild/moderate disease did not suffer from long-term pulmonary sequalae and would not require routine follow-up. COVID-19 survivors with more severe diseases might develop long-term pulmonary sequalae, notably impaired DLCO and radiological findings of mild non-fibrotic ILAs. Persistent fibrotic ILAs, pulmonary fibrosis and pulmonary embolism were rare. Patients with more severe diseases and persistent symptoms or radiological changes should be considered for pulmonary function testing. This could be performed 6 months post-infection, with 6 monthly interval PFTs until resolution. Non-COVID-19 and extrapulmonary causes of abnormal PFTs should also be considered and worked up early.

### Supplementary Information

Supplementary Information 1.

Supplementary Information 2.

## Data Availability

All data generated or analysed during this study are included in this published article (Supplemental excel file).
