# Supplementary material for: Impact of COVID infection on lung function test and quality of life
Source: Sci Rep. 2023 Oct 12;13:17275. doi: 10.1038/s41598-023-43710-w (PMC10570308; doi:10.1038/s41598-023-43710-w)
Supplement: Supplementary file 2 — Supplementary Information 2. [file 41598_2023_43710_MOESM2_ESM.docx]

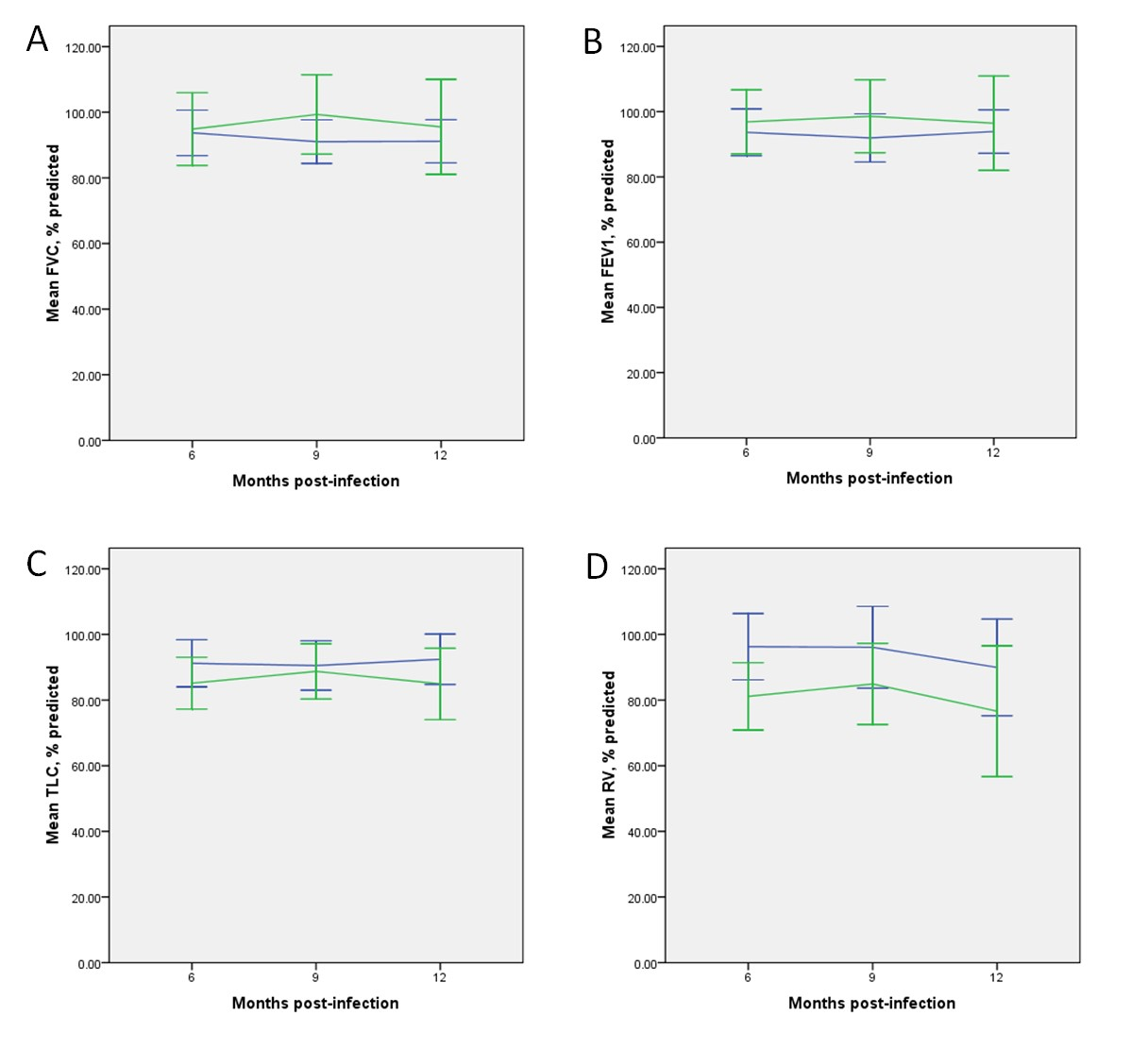


Supplemental figure 1. Plot of disease severity and time based on linear mixed model analysis. The spirometry measurements and lung volumes did not differ between the two disease severity groups. There were also no significant temporal variations in these aspects of lung function. Mild/moderate disease and severe/critical illness were represented by the blue and green lines respectively. Error bars indicated 95% confidence interval and statistical significance was represented by an asterisk. Abbreviations: FVC (forced vital capacity), FEV1 (forced expiratory volume in 1 sec), TLC (total lung capacity), RV (residual volume).


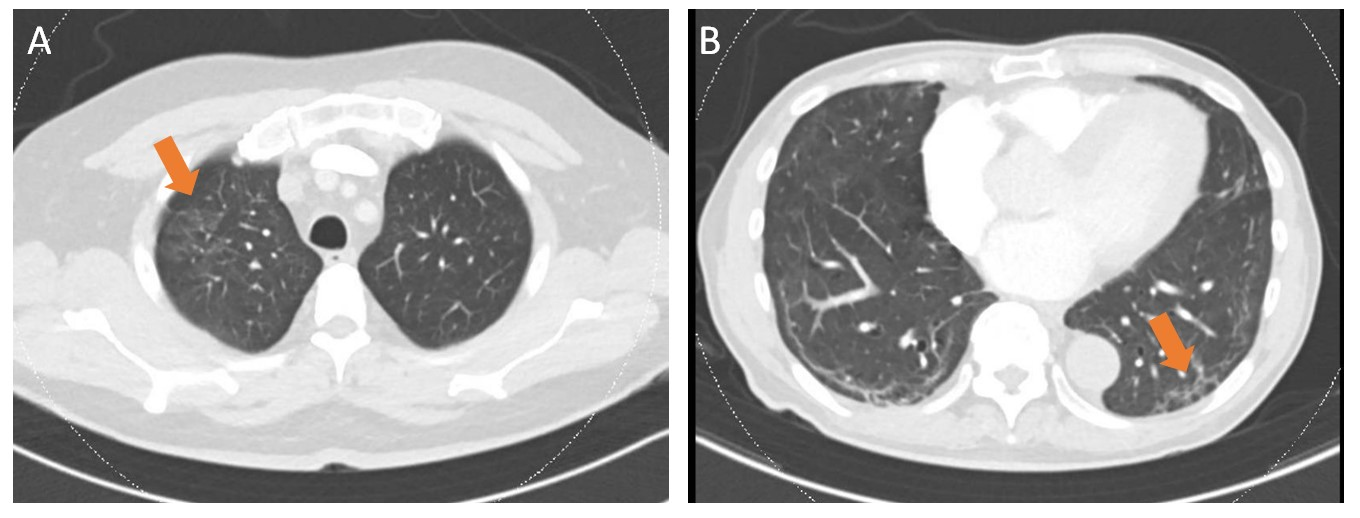


Supplemental figure 2. CT thorax of COVID-19 survivors showing mild (A) ground glass opacities and (B) subpleural reticulations

Supplemental table 1. Characteristics of the patients with DLCO defects

| Patient | Disease severity | | PFT at 18 months | Interval CT finding | Clinical diagnosis |
| --- | --- | --- | --- | --- | --- |
| COVID-001 | Critical illness, complicated by ARDS requiring invasive mechanical ventilation | | Persistent DLCO defect | Non-fibrotic ILA (bilateral subpleural reticulations) | Severe COVID-19 pneumonia |
| COVID-002 | Mild disease | | Normalised | Declined | Mild COVID-19 pneumonia |
| COVID-019 | Critical illness complicated by shock requiring inotropes | | Declined | Declined | Severe COVID-19 pneumonia |
| COVID-022 | Critical illness, complicated by ARDS and shock requiring invasive mechanical ventilation and inotropes | | Normalised | Non-fibrotic ILA (subpleural bands) | Severe COVID-19 pneumonia, morbid obesity |
| COVID-025 | Severe disease | | Normalised | Non-fibrotic ILA (bilateral GGO, subpleural reticulations) | Severe COVID-19 pneumonia |
| COVID-033 | Mild disease | | Persistent DLCO defect | Not ILA, mild subpleural bands | Mild COVID-19 pneumonia, morbid obesity |
| COVID-034 | Critical illness complicated by shock requiring inotropes | | Normalised | Non-fibrotic ILA (bilateral upper lobe reticulations and subpleural blebs) | Severe COVID-19 pneumonia |
| COVID-040 | Mild disease | | Declined | Declined | Mild COVID-19 pneumonia |
| COVID-041 | Severe disease | | Persistent DLCO defect | Normal | Severe COVID-19 pneumonia, morbid obesity |
| COVID-042 | Critical illness, complicated by ARDS requiring invasive mechanical ventilation | | Persistent DLCO defect | Normal | Severe COVID-19 pneumonia, morbid obesity  COPD |
| COVID-047 | Critical illness, complicated by ARDS requiring invasive mechanical ventilation | | Declined | Non-fibrotic ILA (bilateral upper lobe subpleural reticulations) | Severe COVID-19 pneumonia |
| COVID-049 | Mild disease | | Declined | Declined | Mild COVID-19 pneumonia |
| COVID-050 | Severe disease | | Declined | Declined | Severe COVID-19 pneumonia |
| COVID-052 | Mild disease | | Not needed due to non-COVID-19 diagnosis | Declined | Morbid obesity  Fluid overload |
| COVID-055 | Critical illness, complicated by ARDS requiring invasive mechanical ventilation | | Declined | Non-fibrotic ILA (bilateral GGO and reticulations, subpleural cysts) | Severe COVID-19 pneumonia |
|  | |  |  |  |  |

Supplemental table 2. Characteristics of the patients with other PFT defects

| Patient | Disease severity | PFT defect | Interval CT finding | Clinical diagnosis |
| --- | --- | --- | --- | --- |
| COVID-022 | Critical illness, complicated by ARDS and shock requiring invasive mechanical ventilation and inotropes | Restrictive ventilatory defect, impaired DLCO | Non-fibrotic ILA (subpleural bands) | Severe COVID-19 pneumonia, morbid obesity |
| COVID-025 | Severe disease | Restrictive ventilatory defect, impaired DLCO | Non-fibrotic ILA (bilateral GGO, subpleural reticulations) | Severe COVID-19 pneumonia |
| COVID-026 | Mild disease | Restrictive ventilatory defect | Declined | Mild COVID-19 pneumonia, morbid obesity |
| COVID-033 | Mild disease | Restrictive ventilatory defect, impaired DLCO | Not ILA, mild subpleural bands | Mild COVID-19 pneumonia, morbid obesity |
| COVID-034 | Critical illness complicated by shock requiring inotropes | Restrictive ventilatory defect, impaired DLCO | Non-fibrotic ILA (bilateral upper lobe reticulations and subpleural blebs) | Severe COVID-19 pneumonia |
|  |  |  |  |  |
| COVID-037 | Mild disease | Restrictive ventilatory defect | Not needed due to non-COVID-19 diagnosis | Mild COVID-19 pneumonia, morbid obesity |
| COVID-040 | Mild disease | Restrictive ventilatory defect, impaired DLCO | Declined | Mild COVID-19 pneumonia |
| COVID-041 | Severe disease | Restrictive ventilatory defect, impaired DLCO | Normal | Severe COVID-19 pneumonia, morbid obesity |
| COVID-042 | Critical illness, complicated by ARDS requiring invasive mechanical ventilation | Restrictive ventilatory defect, impaired DLCO | Normal | Severe COVID-19 pneumonia, morbid obesity  COPD |
| COVID-047 | Critical illness, complicated by ARDS requiring invasive mechanical ventilation | Restrictive ventilatory defect, impaired DLCO | Non-fibrotic ILA (bilateral upper lobe subpleural reticulations) | Severe COVID-19 pneumonia |
| COVID-049 | Mild disease | Restrictive ventilatory defect, impaired DLCO | Declined | Mild COVID-19 pneumonia |
| COVID-052 | Mild disease | Restrictive ventilatory defect, impaired DLCO | Declined | Morbid obesity  Fluid overload |
| COVID-053 | Critical illness, complicated by ARDS | Restrictive ventilatory defect | Not needed due to non-COVID-19 diagnosis | Severe COVID-19 pneumonia, morbid obesity |
| COVID-004 | Mild disease | Obstructive ventilatory defect | Declined | Mild COVID-19 pneumonia |
| COVID-009 | Mild disease | Obstructive ventilatory defect | Not needed due to non-COVID-19 diagnosis | Asthma |
| COVID-013 | Mild disease | Obstructive ventilatory defect | Declined | Mild COVID-19 pneumonia |
